# Supplementary material for: Redox gated polymer memristive processing memory unit
Source: Nat Commun. 2019 Feb 13;10:736. doi: 10.1038/s41467-019-08642-y (PMC6374435; doi:10.1038/s41467-019-08642-y)
Supplement: Supplementary file 1 — Supplementary Information [file 41467_2019_8642_MOESM1_ESM.pdf]

Redox Gated Polymer Memristive Processing Memory Unit

Bin Zhang[1†], Fei Fan[1†], Wuhong Xue[3,4†], Gang Liu*[2,3], Yubin Fu*[5], Xiaodong Zhuang[2,5], Xiao-Hong Xu[4], Junwei Gu[6], Run-Wei Li[3], and Yu Chen*[1]

[1]Key Laboratory for Advanced Materials, Institute of Applied Chemistry, School of Chemistry and Molecular Engineering, East China University of Science and Technology, Shanghai 200237, China

[2]School of Chemistry and Chemical Engineering, Shanghai Jiao Tong University, Shanghai 200240, China

[3]CAS Key Laboratory of Magnetic Materials and Devices, Ningbo Institute of Materials Technology and Engineering, Chinese Academy of Sciences, Ningbo, Zhejiang 315201, China

[4]Key Laboratory of Magnetic Molecules and Magnetic Information Materials of Ministry of Education, School of Chemistry and Materials Science, Shanxi Normal University, Linfen, Shanxi 041004, China

[5]Center for Advancing Electronics Dresden (cfaed) & Department of Chemistry and Food Chemistry, Technische Universität Dresden, Dresden 01062, Germany

[6]Shaanxi Key Laboratory of Macromolecular Science and Technology, Department of Applied Chemistry, School of Science, Northwestern Polytechnical University, Xi' an, Shaanxi, 710072, China.

All correspondence and request for materials should be addressed to: chentangyu@yahoo.com (Prof Yu Chen), liug@nimte.ac.cn (Prof. Gang Liu), yubin.fu@tu-dresden.de (Dr. Yubin Fu)

[†]These authors contributed equally to this work.

Supplementary Figures

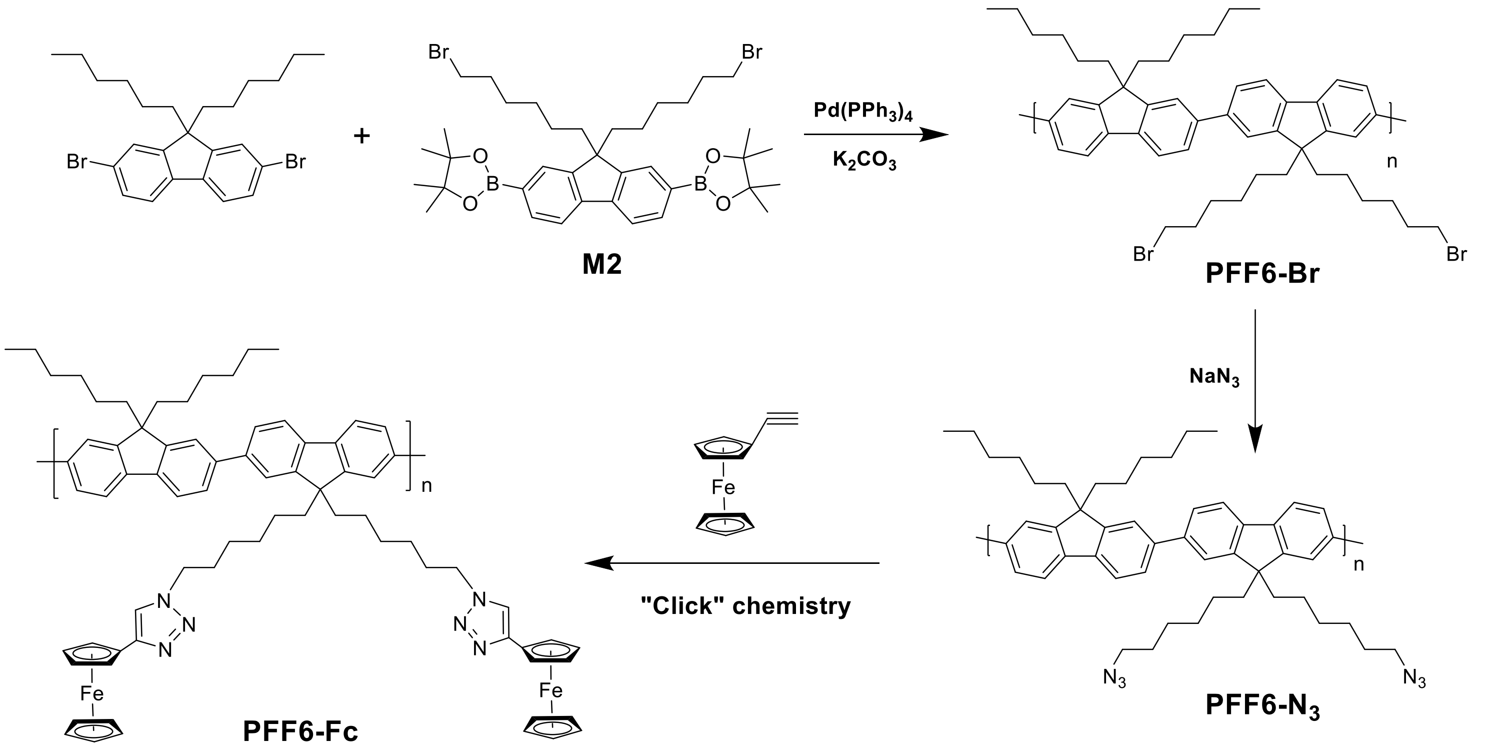

Supplementary Figure 1. Synthetic route of PFF6-Fc.

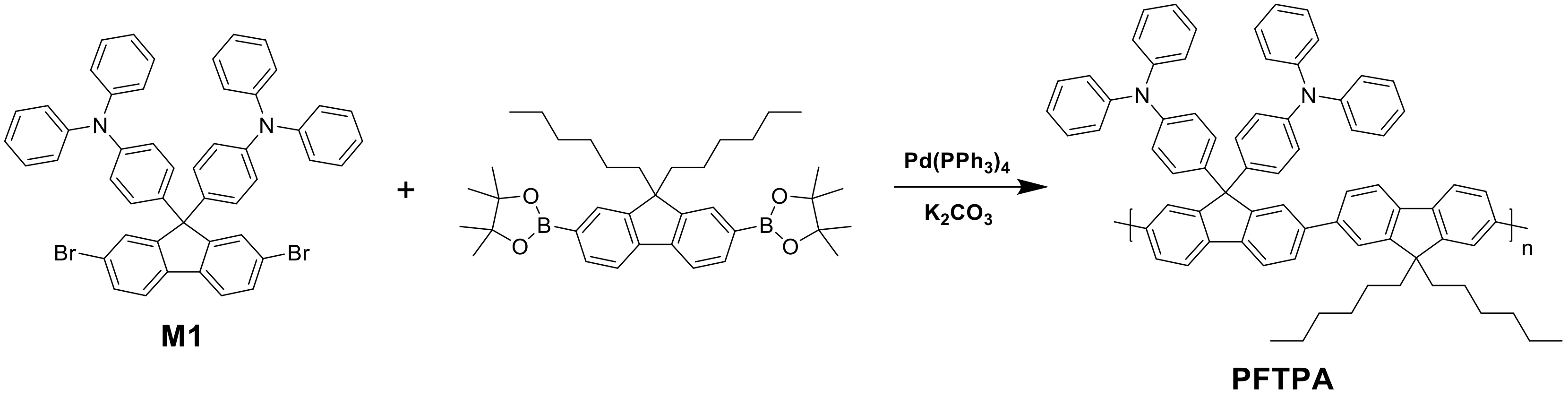

Supplementary Figure 2. Synthetic route of PFTPA.

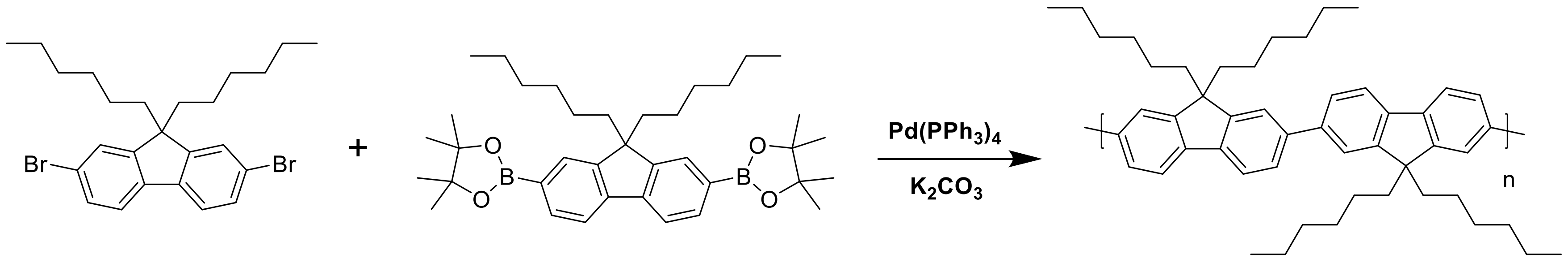

PFF6

Supplementary Figure 3. Synthetic route of PFF6.

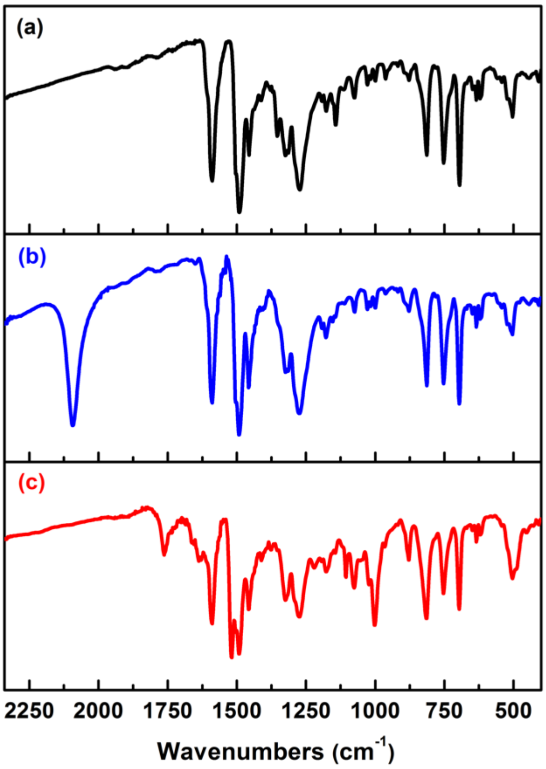

Supplementary Figure 4. FTIR spectra of the polymers. (a) PFTPA-Br, (b) PFTPA-N_3, and (c) PFTPA-Fc, respectively.

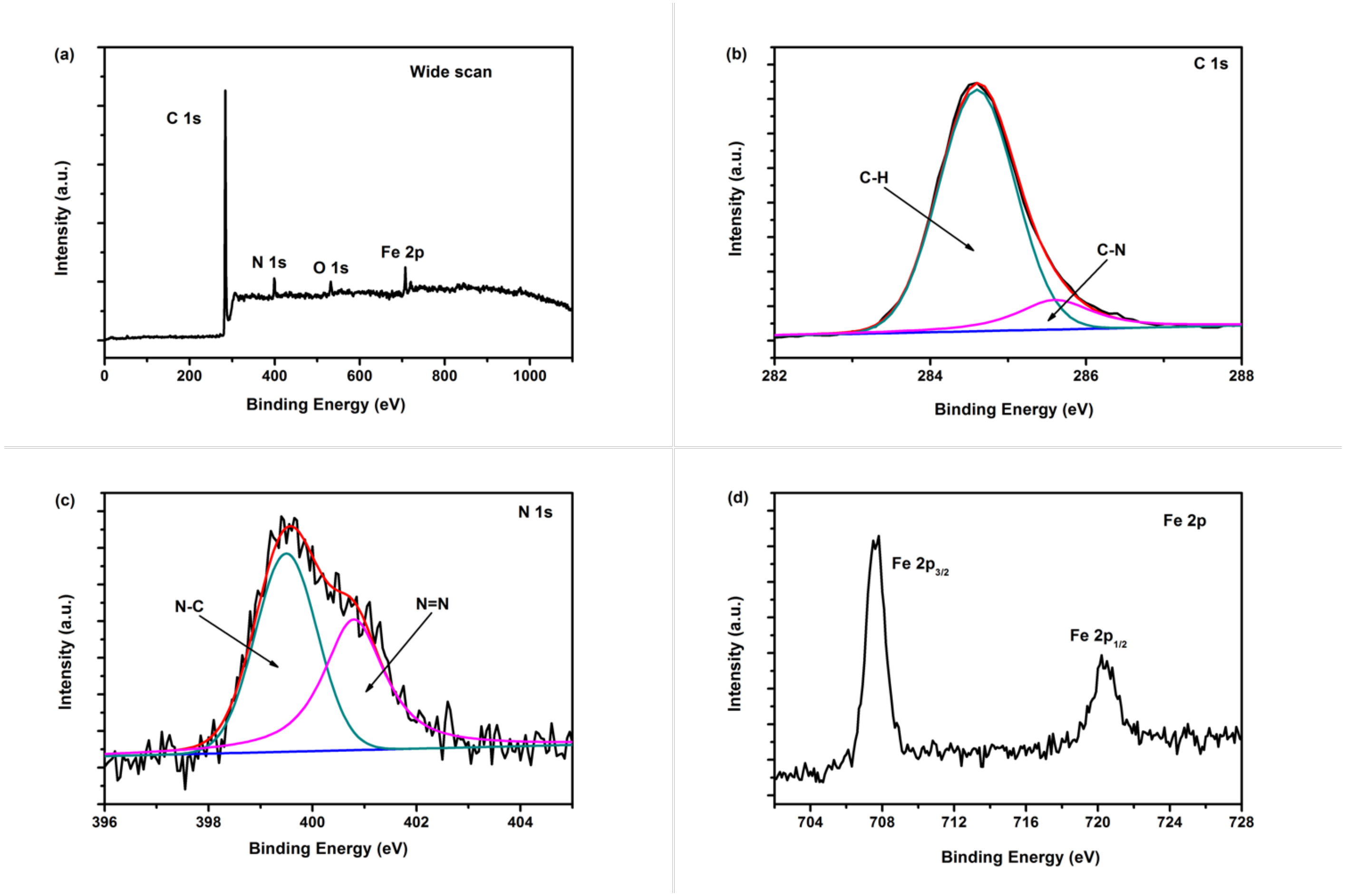

Supplementary Figure 5. Core-level X-ray photoelectron spectra (XPS) of PFTPA-Fc. (a) wide-scan spectra, (b) C 1s core-level spectra, (c) N 1s core-level spectra, and (d) Fe 2p spectra of PFTPA-Fc, respectively.

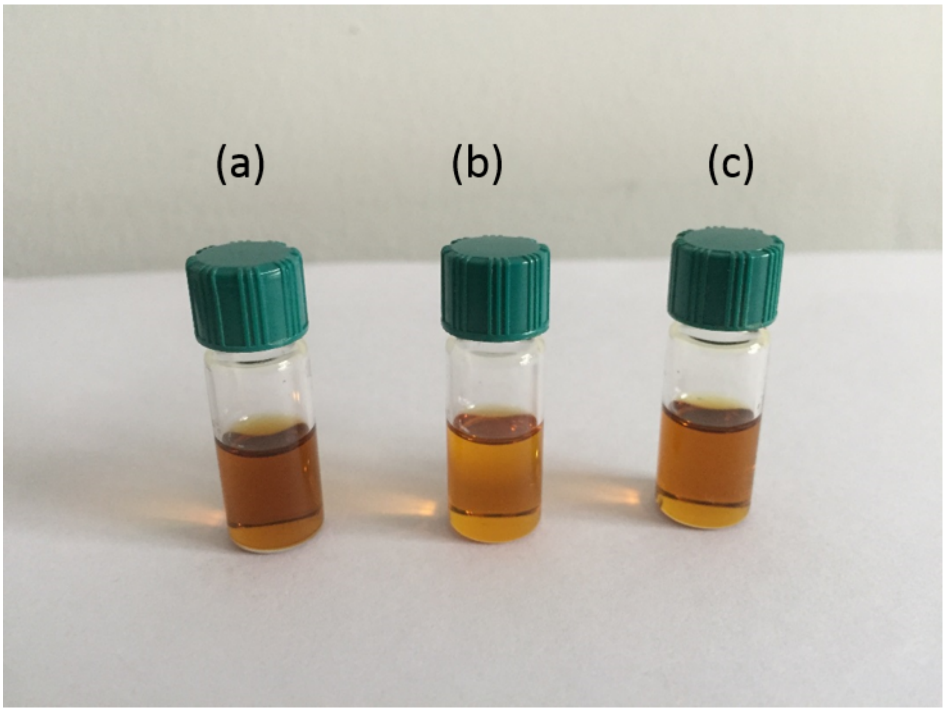

Supplementary Figure 6. Image of PFTPA-Fc dissolved in different solvents. Solutions in (a) THF, (b) toluene and (c) DMF. The concentrations are all 10 mg/mL in these solutions. The image was taken with a digital camera.

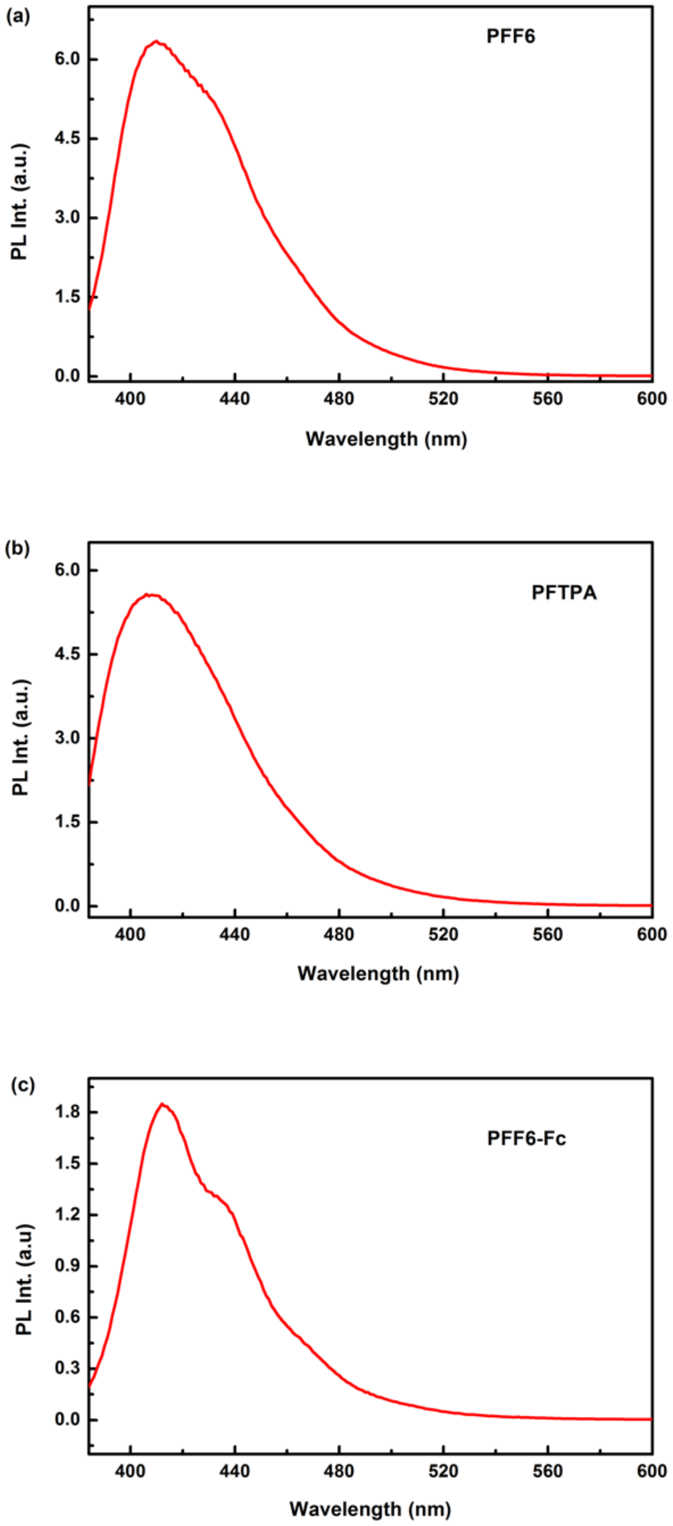

Supplementary Figure 7. Fluorescence spectra of the polymers. (a) PFF6 containing no TPA or Fc moieties, (b) PFTPA containing only TPA pendant moieties and (c) PFF6-Fc containing only Fc pendant moieties, respectively.

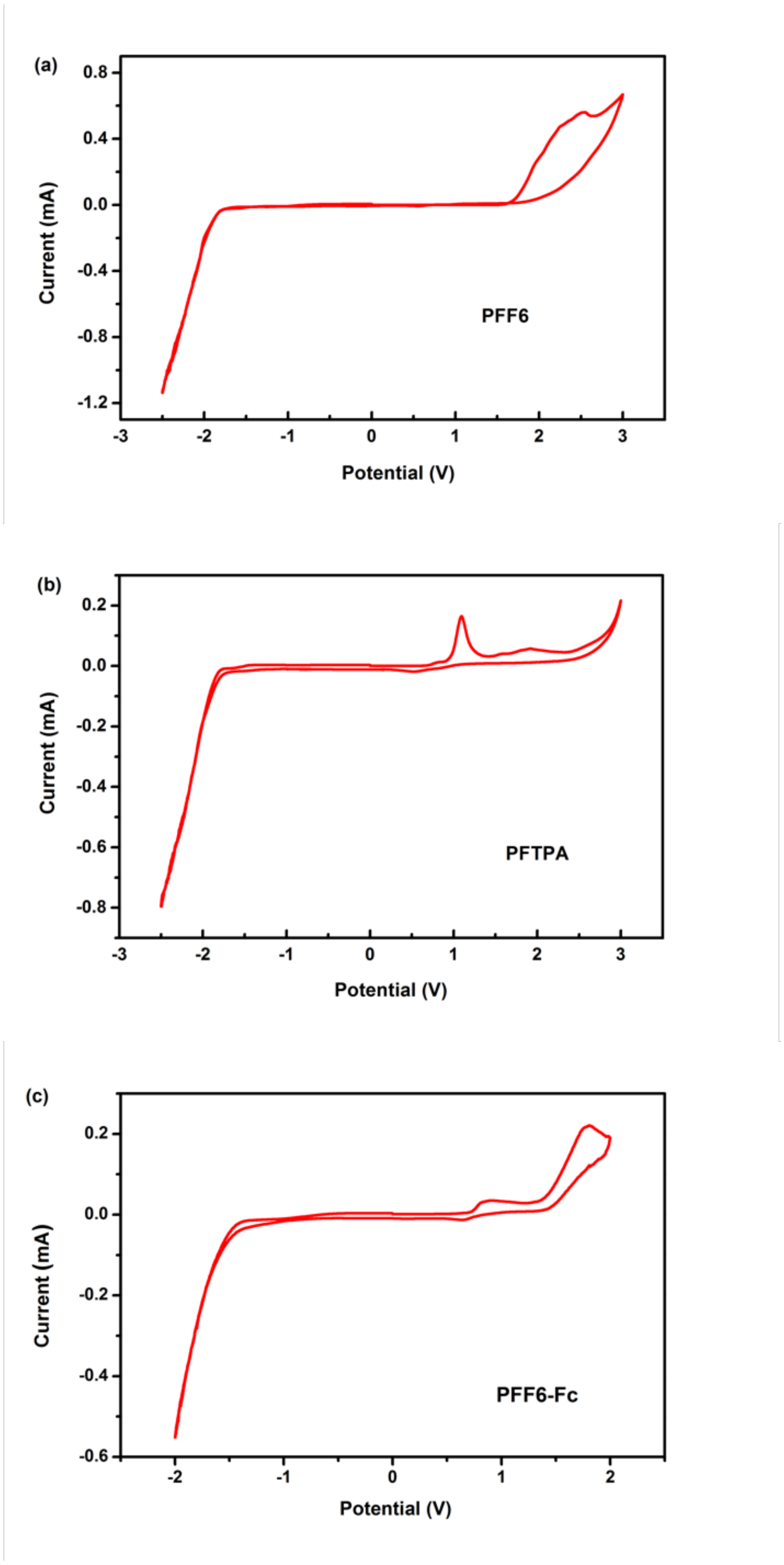

Supplementary Figure 8. Cyclic voltammetry spectra of the polymers. (a) PFF6 containing no TPA or Fc moieties, (b) PFTPA containing only TPA pendant moieties and (c) PFF6-Fc containing only Fc pendant moieties, respectively.

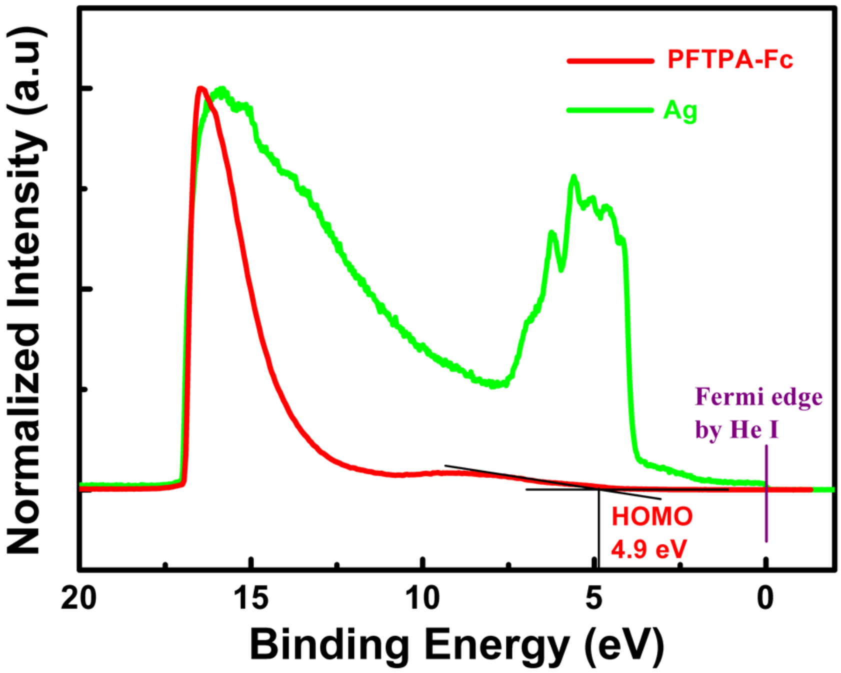

Supplementary Figure 9. Ultra-violet photoelectron spectrum (UPS) of PFTPA-Fc. The HOMO level of the polymer can be measured from the UPS spectrum edge as ~ 4.90 eV, which is similar to that obtained from the cyclic voltammetry measurement (5.03 eV).

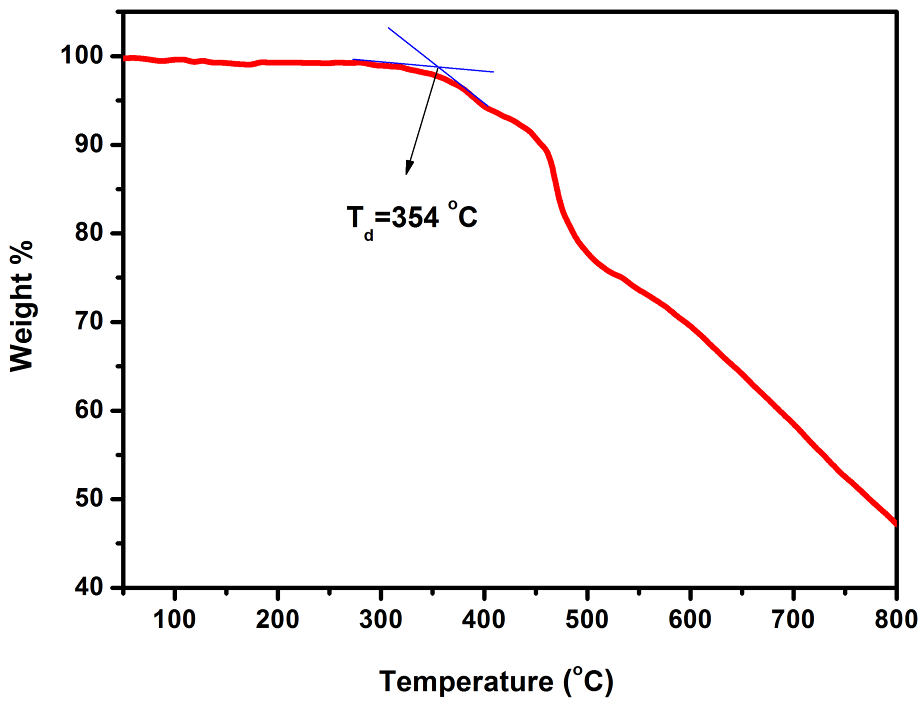

Supplementary Figure 10. TGA profile of the polymer PFTPA-Fc. Experiments were carried out in highly purified nitrogen atmosphere.

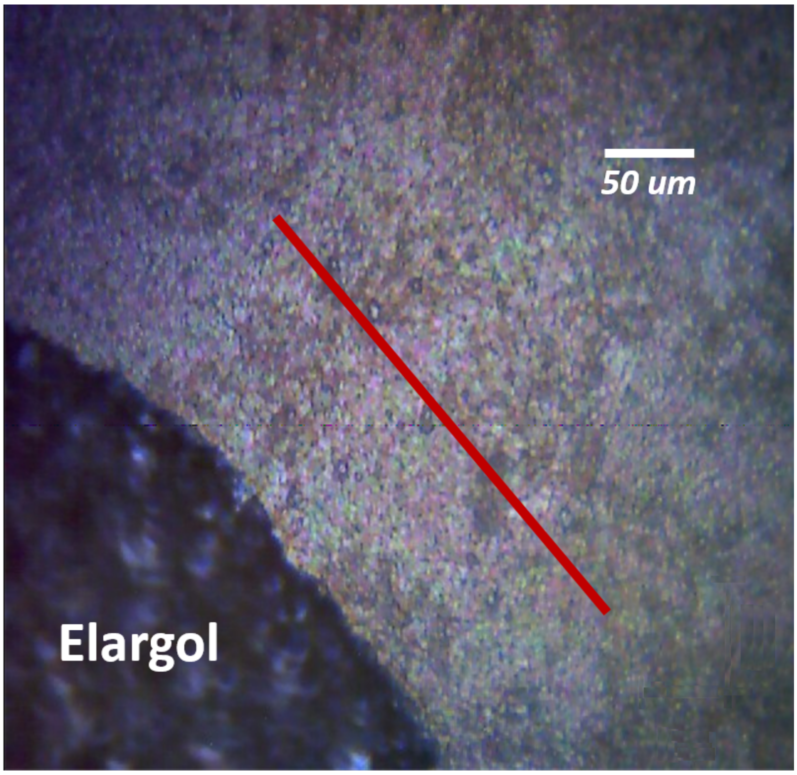

Supplementary Figure 11. Optical image of device for spectrum measurements. The red line shows the scanning area on top of the 500 μm diameter ITO electrode of the ITO/PFTPA-Fc/Pt device for *in-situ* fluorescence measurements. The transparent ITO electrode cannot be visualized with the optical microscope while Elargol is used for electrode wire-leading.

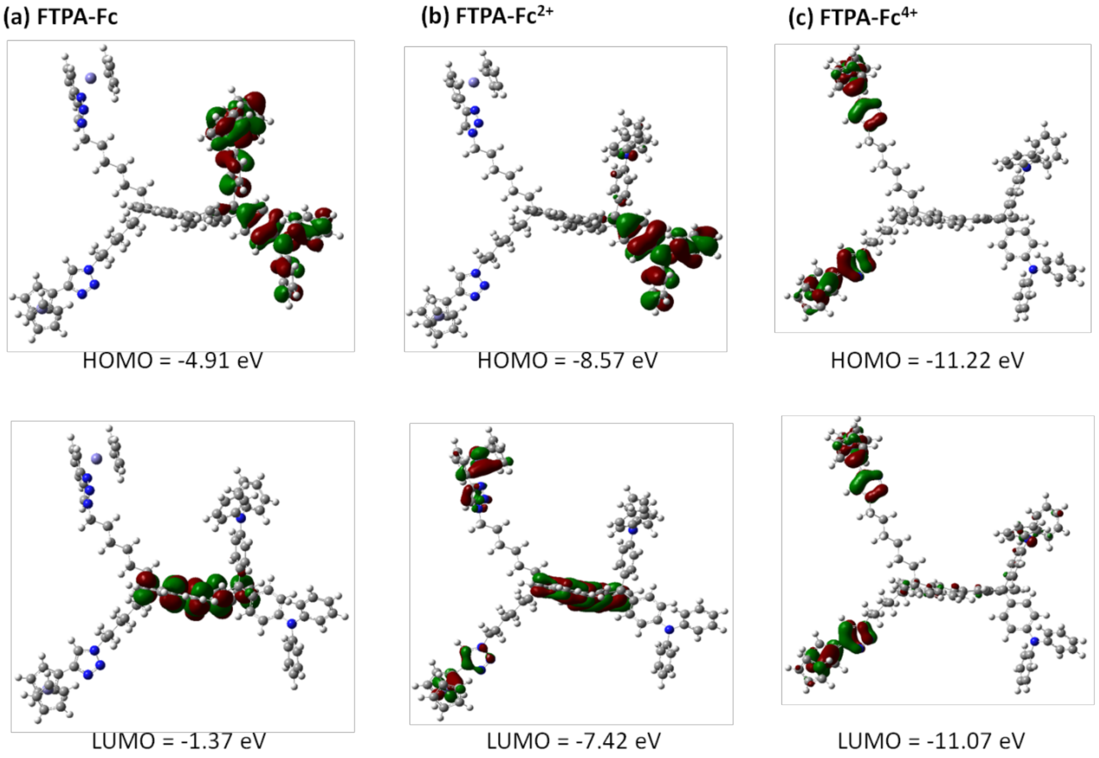

Supplementary Figure 12. Molecular orbitals of the FTPA-Fc repeating unites. Simulated HOMO and LUMO profiles in different oxidation states of (a) prinstine FTPC-Fc, (b) FTPA-Fc^{2+} and (c) FTPA-Fc^{4+}, respectively. The grey, white, blue and light purple spheres represent carbon, hydrogen, nitrogen and iron atoms, respective-ly.

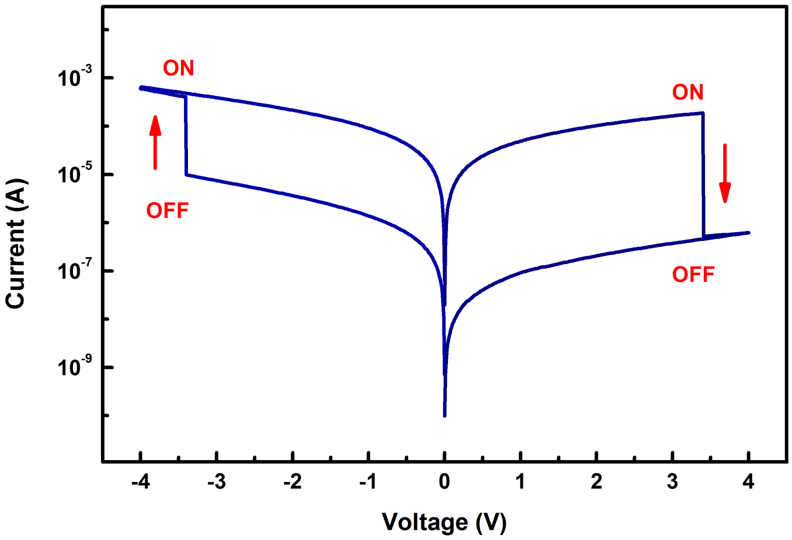

Supplementary Figure 13. Current-voltage characteristics of the ITO/PFTPA-Fc/Pt device recorded in the ±4 V voltage range.

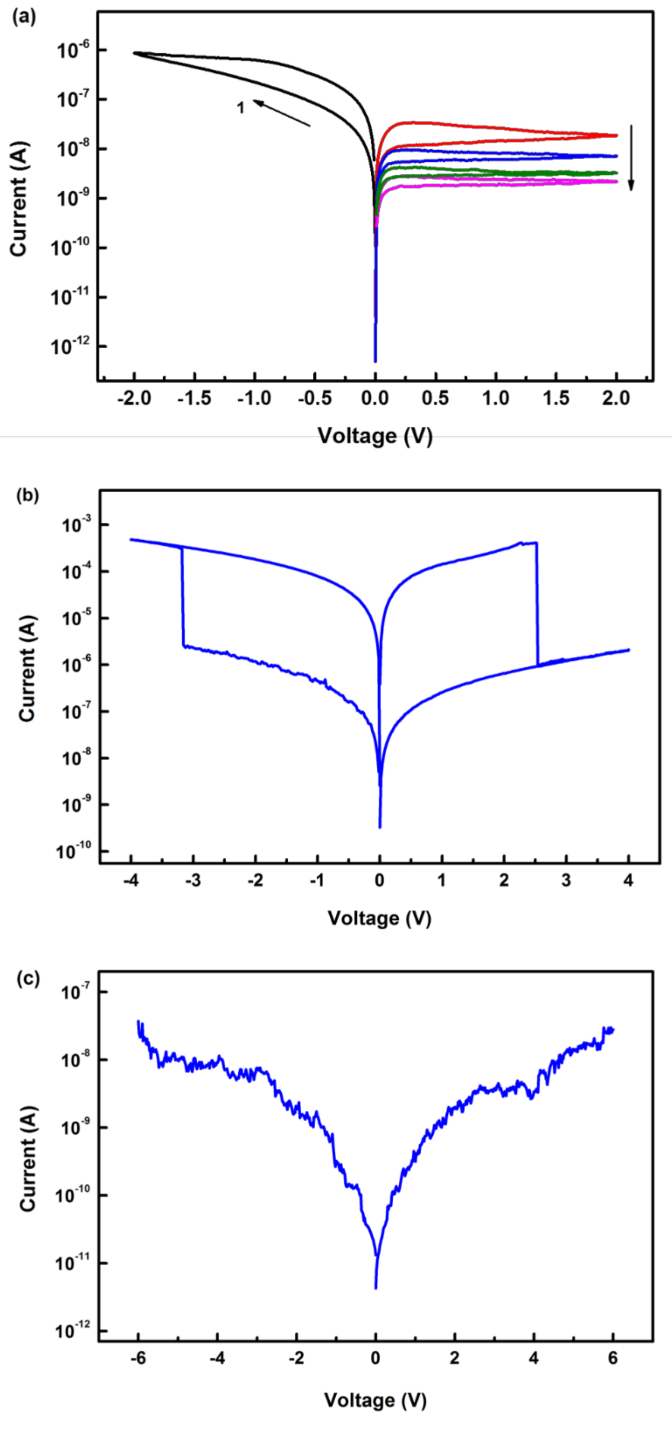

Supplementary Figure 14. Current-voltage characteristics of the polymer devices. I-V curves of the devices constructed from (a) PFF6-Fc, (b) PFTPA and (c) PFF6, re-spectively.

Supplementary Notes

Supplementary Note 1: Synthesis and Characterization of Monomers and Polymers

All chemicals were purchased from Aldrich and used without further purification. Organic solvents were purified, dried, and distilled under dry nitrogen. 2,7-dibromo-9,9-di(triphenylamine)fluorene (**M1**) and bis[9,9-bis(6-bromohexyl)-fluo renyl]-4,4,5,5- tetramethyl-[1.3.2]dioxaborolane (**M2**) were synthesized according to the methods reported in the literatures.[3,4]

2,7-dibromo-9,9- di(triphenylamine)fluorene (**M1**): ^{1}H NMR ($CDCl_3$, 400 MHz): δ/ppm=7.56 (d, 2H), 7.51 (d, 2H), 7.46 (dd, 2H), 7.22 (m, 8H), 6.99 (m, 20H). ^{13}C NMR (100 MHz, CDCl3): δ/ppm=143.1, 139.0, 131.4, 129.3, 126.7, 123.6, 123.4, 122.2, 121.9, 116.2, 109.3.

bis[9,9-bis(6-bromohexyl)-fluo renyl]-4,4,5,5- tetramethyl-[1.3.2]dioxaborolane (**M2**): ^{1}H NMR ($CDCl_3$, 400 MHz): δ/ppm=7.83-7.70 (m, 6H), 3.27-3.23 (t, 4H), 2.03-1.98 (m, 4H), 1.60 (q, 4H), 1.39 (s, 24H), 1.17-1.12 (q, 4H), 1.07-1.01 (q, 4H), 0.57-0.52 (m, 4H). ^{13}C NMR (100 MHz, CDCl3): δ/ppm=150.1, 143.9, 133.8, 128.8, 119.5, 83.8, 55.1, 39.9, 34.1, 32.7, 29.1, 27.7, 23.6.

Synthesis of poly[9,9-di(triphenylamine)fluorine-*alt*-9,9-bis(6-bromohexyl)fluorene] (**PFTPA-Br**) *via* Suzuki coupling polymerization: to a 25 mL Schlenk tube charged with **M1** (810 mg, 1 mmol), **M2** (744 mg, 1 mmol), and potassium carbonate (552 mg, 4 mmol) was added tetrakis(triphenylphosphine)palladium (8 mg) in a glove-box. Degassed toluene (10 mL) and water (2 mL) was added into the mixture by syringe (**Supplementary Figure 1**). Then the mixture was stirred at 80 °C under nitrogen atmosphere for 36 h. The crude product was

extracted with chloroform at room temperature. The combined organic layers were washed with water and brine, respectively, and then dried over anhydrous sodium sulfate. The collected precipitate from methanol was further purified by Soxhlet extraction with acetone. The polymer was then dried in vacuum at 50 °C overnight to give a yellowish solid. **PFTPA-Br:** ^{1}H NMR ($CDCl_3$, 400 MHz): δ/ppm=7.90-7.55 (m, 12H), 7.20-6.90 (m, 28H), 3.22 (br, 4H), 2.06 (br, 4H), 1.35- 1.08 (m, 12H), 0.76 (br, 4H). ^{13}C NMR (100 MHz, $CDCl_3$): δ/ppm=147.6, 129.2, 127.3, 124.6, 122.8, 29.3, 27.7, 24.9. GPC (THF as eluent): Mn = 17 kDa, PDI = 1.75.

Synthesis of poly[9,9-di(triphenylamine)fluorine-*alt*-9,9-bis(6-azidohexyl)fluorene] (**PFTPA-N$_3$**): a reported procedure was employed for the synthesis of PFTPA-N$_3$ (**Supplementary Figure 1**).[5] Typically, PFTPA-Br (570 mg, 0.5 mmol of PFTPA-Br repeat unit) was dissolved in 20 mL of tetrahydrofuran (THF). NaN_3 (130 mg, 2 mmol) was dissolved in 20 mL of dimethyl formamide (DMF). Then the PFTPA-Br and NaN_3 solutions were mixed in a 100 mL round-bottom flask and stirred at 70 °C for 24 h. After removing THF on a rotary evaporator, the residual solution was filtered and then dialyzed against distilled water for 3 days. **PFTPA-N$_3$** was dried using a lyophilizer and obtained as a pale yellow powder. **PFTPA-N$_3$:** ^{1}H NMR ($CDCl_3$, 400 MHz): δ/ppm= 7.89-7.41 (m, 12H), 7.27-6.83 (m, 28H), 3.08 (br, 4H), 2.05 (br, 4H), 1.40-1.13 (m, 12H), 0.77 (br, 4H). ^{13}C NMR (100 MHz, $CDCl_3$): δ/ppm=147.6, 129.2, 128.9, 124.5, 122.9, 51.3, 28.7, 26.3. GPC (THF as eluent): Mn = 16 kDa, PDI = 1.83.

Synthesis of the ferrocene functionalized polyfluorene: **PFTPA-Fc** was synthesized *via* azide-alkyne “Click” chemistry (**Figure 1**). Briefly, **PFTPA-N$_3$** (214 mg, 0.4 mmol azide

moieties), ethynylferrocene (126 mg, 0.6 mmol alkynyl moieties), and THF (20 mL) were introduced into a 50 mL Schlenk tube. The reaction mixture was degassed with purified argon for 20 min. PMDETA (84 μL, 0.4 mmol) and CuBr (72 mg, 0.5 mmol) were then added to the reaction mixture. The reaction tube was sealed under an argon atmosphere, and the mixture was then stirred at 60 °C for 24 h. After the reaction, the mixture solution was added into stirred methanol. The collected solid from methanol was then dissolved in THF and dialyzed (MW cutoff, 1 kDa) against deionized water. The final product **PFTPA-Fc** was obtained by freeze-drying. **PFTPA-Fc:** ^{1}H NMR ($CDCl_3$, 400 MHz): δ/ppm= 7.90-7.50 (m, 14H), 7.23-6.93 (m, 28H), 4.64 (br, 4H), 4.23-3.99 (m, 18H), 2.03 (br, 4H), 1.38-1.10 (m, 12H), 0.74 (br, 4H). ^{13}C NMR (100 MHz, $CDCl_3$): δ/ppm=152.0, 131.3, 130.2, 127.1, 126.3, 122.8, 69.5, 68.6, 66.5, 29.6, 26.4. GPC (THF as eluent): Mn = 22 kDa, PDI = 1.95. The complete disappearance of the signals at the chemical shift of 3.08 (br, 4H) (as in **PFTPA-N$_3$**) suggests a full consumption of the azide moieties during the "Click" reaction with excess amount of ethynylferrocene, which is in good agreement with the disappearance of azide transmission peak at the wavenumber of 2097 cm^{-1} in the FTIR spectra of the **Supplementary Figure 4b** and **4c**. Furthermore, the newly appearing signals at the chemical shift of 4.64 (br, 4H) and the 4:18 atomic ratio of the signals at the chemical shifts of 4.64 (br, 4H) and 4.23-3.99 (m, 18H) indicate the 100% grafting of the ferrocene groups. As such, it can be confirmed that each repeating unit of the PFTPA-Fc polymers contains two ferrocene unities as shown in **Figure 1**.

Synthesis of **PFF6-Fc**: **PFF6-Fc** was prepared through Suzuki coupling polymerization and "Click" chemistry (**Supplementary Figure 1**). The detailed experimental procedures

were the same as the synthesis of **PFTPA-Fc**, except for 2,7-dibromo-9,9-dihexyl-fluorene and **M2** were used as the initial monomers. **PFF6-Fc:** ^{1}H NMR ($CDCl_3$, 400 MHz): δ/ppm= 7.83-7.31 (m, 14H), 4.68 (br, 4H), 4.26-4.02 (m, 18H), 2.05 (br, 8H), 1.44-1.12 (m, 24H), 0.78 (br, 14H). ^{13}C NMR (100 MHz, $CDCl_3$): δ/ppm=147.8, 141.0, 128.5, 126.6, 122.1, 69.5, 68.5, 66.7, 50.2, 31.5, 29.6, 22.6. GPC (THF as eluent): Mn = 17 kDa, PDI = 1.78.

Synthesis of **PFTPA**: To a 25 mL Schlenk tube charged with **M1** (810 mg, 1 mmol), 9,9-dihexylfluorene-2,7-bis(4,4,5,5-tetramethyl-1,3,2-dioxaborolan) (586 mg, 1 mmol), and potassium carbonate (552 mg, 4 mmol) was added tetrakis(triphenyl phosphine)palladium (8 mg) in a glove-box (**Supplementary Figure 2**). Degassed toluene (10 mL) and water (2 mL) was added into the mixture by syringe. Then the mixture was stirred at 80 °C under nitrogen atmosphere for 36 h. The crude product was extracted with chloroform at room temperature. The combined organic layers were washed with water and brine, respectively, and then dried over anhydrous sodium sulfate. The collected precipitate from methanol was further purified by Soxhlet extraction with acetone. The polymer was then dried in vacuum at 50 °C overnight to give a light yellow solid. **PFTPA:** ^{1}H NMR ($CDCl_3$, 400 MHz): δ/ppm= 7.42-7.89 (m, 12H), 6.81-7.20 (m, 28H), 2.08 (br, 4H), 1.02-1.38 (m, 12H), 0.64-0.78 (m, 10H). ^{13}C NMR (100 MHz, $CDCl_3$): δ/ppm=147.7, 146.7, 134.6, 129.3, 127.3, 124.6, 122.8, 121.5, 55.2, 40.1, 31.4, 29.6, 24.9, 23.6, 22.5, 14.0. GPC (THF as eluent): Mn = 13 kDa, PDI = 1.72.

Synthesis of **PFF6**: To a 25 mL Schlenk tube charged with 2,7-dibromo-9,9- dihexyl-fluorene (492 mg, 1 mmol), 9,9-dihexylfluorene-2,7-bis(4,4,5,5-tetramethyl- 1,3,2-dioxa borolan) (586 mg, 1 mmol), and potassium carbonate (552 mg, 4 mmol) was added

tetrakis(triphenylphosphine)palladium (8 mg) in a glove-box (**Supplementary Figure 3**). Degassed toluene (10 mL) and water (2 mL) was added into the mixture by syringe. Then the mixture was stirred at 80 °C under nitrogen atmosphere for 36 h. The crude product was extracted with chloroform at room temperature. The combined organic layers were washed with water and brine, respectively, and then dried over anhydrous sodium sulfate. The collected precipitate from methanol was further purified by Soxhlet extraction with acetone. The polymer was then dried in vacuum at 50 °C overnight to give a white solid. **PFF6:** ^{1}H NMR ($CDCl_3$, 400 MHz): δ/ppm= 7.30-7.82 (m, 6H), 2.12 (br, 4H), 1.01-1.37 (m, 12H), 0.65-0.79 (m, 10H). ^{13}C NMR (100 MHz, $CDCl_3$): δ/ppm=147.6, 131.8, 128.6, 122.1, 40.3, 31.4, 29.6, 24.9, 23.7, 22.6, 14.0. GPC (THF as eluent): Mn = 11 kDa, PDI = 1.56.

Supplementary Note 2: Fourier Transformed Infrared (FTIR) Spectra of Polymers

FTIR spectra of the PFTPA-Br, PFTPA-N_3 and PFTPC-Fc are shown in **Supplementary Figure 4**. Comparing the spectra of PFTPA-Br and PFTPA-N_3, we can find that the main absorption bands of the two spectra are roughly the same. However, a new peak at about 2094 cm^{-1} in the spectrum of PFTPA-N_3 can be seen obviously, which is assigned to the stretching vibration of the azide groups. Upon the reaction between the PFTPA-N_3 and acetylene ferrocene through "click chemistry", the absorption peak at the wavenumber of 2094 cm^{-1} is vanished in the spectrum of the as-synthesized PFTPA-Fc. It is also noteworthy that three new peaks at 1758 cm^{-1}, 1520 cm^{-1} and 1002 cm^{-1} appear, which are correlated to the vibration of the cyclopentadienyl on ferrocene moieties, demonstrating that PFTPA-Fc has been successfully synthesized.

Supplementary Note 3: XPS spectra and Composition of PFTPA-Fc

The chemical composition of PFTPA-Fc was further analyzed by X-ray photoelectron spectroscopy (XPS) measurements. The wide scan, C 1s, N 1s and Fe 2p core-level spectra of the polymer are shown in **Supplementary Figure 5(a)** to **5(d)**, respectively. The presence of the amine (285.6 eV and 399.5 eV) N 1s components corresponds to the triphenylamine moieties in the pendants, whereas the N=N species with the binding energy of 400.8 eV is coming from the "Click" linking ligand. The Fe 2p spectrum of PFTPA-Fc with doublet peaks at the binding energies of 707.6 eV and 720.4 eV is characteristics of Fe^{2+} ions of the ferrocene pendants.

Supplementary Note 4: Fluorescence Spectra of Polymers

The fluorescence spectra of PFTPA-Fc, PFF6, PFTPA and PFF6-Fc were all measured under the same conditions, e.g. recorded in diluted toluene solutions and excited at the wavelength of 365 nm. The fluorescence spectrum of PFF6 shows sharp emission at about 409 nm, which can be attributed to the monomer fluorescence of the polymer backbone (**Supplementary Figure 7a**). Minor and broad emission shoulders are also observed at longer wavelength region. For PFTPA the major emission of the polymer backbone locates at 408 nm (**Supple-mentary Figure 7b**). In comparison, PFF6-Fc shows obvious emission shoulder at the 435 nm (**Supplementary Figure 7c**), indicating that the emission shoulder of PFTPA-Fc at 438 nm clearly confirms the successful linking of ferrocene moieties onto the polymer.

Supplementary Note 5: Calculation of Molecular Orbital Levels and Band Gaps

The electrochemical properties of the copolymers are explored in the cyclic voltammetry spectra as plotted in **Figure 2b** and **Supplementary Figure 8**. Due to the difference in the chemical structures, the four polymers of PFF6, PFTPA, PFF6-Fc and PFTPA-Fc exhibit different oxidation profiles, with their onset oxidation potential ranging from 1.68 V, 0.95 V, 0.67 V to 0.61 V, respectively. The molecular orbital energy levels, in particular the highest occupied molecular orbital (HOMO) levels of the homo- and copolymers, can be directly deducted from the equation $HOMO=-(E_{ox}(onset)+4.8-E_{FOC})$,[6] where 4.8 is the reference energy level of ferrocene (FOC) and E_{FOC} is the onset oxidation potential of FOC *vs.* Ag/AgCl reference electrode (0.38 V, as measured by cyclic voltammetry). Therefore, the HOMO level of PFF6, PFTPA, PFF6-Fc and PFTPA-Fc are -6.10 eV, -5.37 eV, -5.09 eV and -5.03 eV, respectively. The lowest unoccupied molecular orbital (LUMO) energy levels are calculated from the difference between the HOMO levels and energy band gaps derived from optical absorption spectra, according to the equations $E_g=hc/\lambda_{edge}$ and $LUMO=HOMO+E_g$, where h is the Planck constant (6.63×10^{-34} m^2kg/s) and c is the speed of light (3×10^8 m/s). The UV-Visible absorption edge of PFTPA-Fc is ~ 420 nm (**Figure 2a**) while its LUMO level is -2.07 eV.

Supplementary Note 6: Weibull Analysis of Device Switching Parameters

Weibull distribution is employed to evaluate the uniformity of the resistive switching parameters of the PFTPA-Fc device by the Equation $F(x) = 1-\exp(-(x/x_0)^k)$, where x and x_0 are random variable and scale parameter of the distribution of x, respectively. k and F are Weibull exponent and the cumulative probability for finding the random variable (herein the device current in different stages) blow x relative to a scale parameter x_0.

Supplementary References

1. Park, N.-G. Perovskite Solar Cells: An Emerging Photovoltaic Technology. *Mater. Today* **18**, 65-72 (2015).

2. Ishii, H., Sugiyama, K., Ito, E. & Seki, K. Energy level alignment and interfacial electronic structures at organic/metal and organic/organic interfaces. *Adv. Mater.* **11**, 605-625 (1999).

3. Ego, C., Grimsdale, A. C., Uckert, F., Yu, G., Srdanov, G. & Mullen, K. Triphenylamine-Substituted Polyfluorene-A Stable Blue-Emitter with Improved Charge Injection for Light-Emitting Diodes. *Adv. Mater.* **14**, 809-811 (2002).

4. Liu, B. & Bazan, G. C. Synthesis of cationic conjugated polymers for use in label-free DNA microarrays. *Nat Protoc* **1**, 1698-1702 (2006).

5. Zhou, L., Geng, J., Wang, G., Liu, J. & Liu, B. A water-soluble conjugated polymer brush with multihydroxy dendritic side chains. *Polym. Chem.* **4**, 5243-5251 (2013).

6. Wang, K.-L., Liu, G., Chen, P.-H., Pan, L. & Tsai, H.-L. Structural effect on controllable resistive memory switching in donor–acceptor polymer systems. *Org. Electron.* **15**, 322-336 (2014).
